# Constructing criminality: a thematic analysis of national news media reporting on self-managed abortion criminalisation in the United States

**DOI:** 10.1080/26410397.2025.2572890

**Published:** 2025-10-15

**Authors:** Hayley V. McMahon, Naya Pearce, Alexis J. Smith, India C. Stevenson, Grace E. Howard, Sara K. Redd, Whitney S. Rice

**Affiliations:** a Doctoral Candidate, Department of Behavioral, Social, and Health Education Sciences, Emory University Rollins School of Public Health, Atlanta, GA, USA; Doctoral Fellow, The Center for Reproductive Health Research in the Southeast (RISE), Emory University Rollins School of Public Health, Atlanta, GA, USA.; b Graduate Student, Department of Behavioral, Social, and Health Education Sciences, Emory University Rollins School of Public Health, Atlanta, GA, USA; Graduate Research Assistant, The Center for Reproductive Health Research in the Southeast (RISE), Emory University Rollins School of Public Health, Atlanta, GA, USA; c Doctoral Candidate, Department of Behavioral, Social, and Health Education Sciences, Emory University Rollins School of Public Health, Atlanta, GA, USA; d Graduate Student, Department of Behavioral, Social, and Health Education Sciences, Emory University Rollins School of Public Health, Atlanta, GA, USA; Graduate Research Assistant, The Center for Reproductive Health Research in the Southeast (RISE), Emory University Rollins School of Public Health, Atlanta, GA, USA; e Associate Professor, Department of Justice Studies, San José State University, San José, CA, USA; f Assistant Professor, Department of Behavioral, Social, and Health Education Sciences, Emory University Rollins School of Public Health, Atlanta, GA, USA; Director of Research Translation, The Center for Reproductive Health Research in the Southeast (RISE), Emory University Rollins School of Public Health, Atlanta, GA, USA; g Assistant Professor, Department of Behavioral, Social, and Health Education Sciences, Emory University Rollins School of Public Health, Atlanta, GA, USA; Director, The Center for Reproductive Health Research in the Southeast (RISE), Emory University Rollins School of Public Health, Atlanta, GA, USA

**Keywords:** self-managed abortion, abortion stigma, reproductive justice, criminalisation, policing pregnancy, news media

## Abstract

Self-managed abortion (SMA) is prevalent in the United States (US) and has become increasingly common since the 2022 Supreme Court ruling in *Dobbs v. Jackson Women’s Health Organization* that allowed states to enact early abortion bans. Although SMA is legal in nearly every US state, the number of people who have been arrested for alleged participation in SMA has continued to grow in recent years. Reproductive justice advocates have identified news media portrayals of these cases as a likely contributor to adverse legal outcomes for criminalised individuals; however, researchers have not yet investigated this. Therefore, we aimed to qualitatively assess the ways in which national news media outlets depict instances of SMA criminalisation. We identified 41 articles published by the top five national newspapers between 2000 and 2023 and conducted a reflexive thematic analysis. Our analysis revealed concerning patterns in news media coverage, in which journalists frequently personified the fetus as a victim while vilifying the criminalised person, undermined the character of the criminalised person through selected personal histories, and promoted the presumption of guilt by treating biased police statements as objective accounts. These findings contribute to the literature by describing the ways in which reproductive injustice is legitimised through news media and provide empirical support for reproductive justice advocates’ calls for more responsible journalism in news media coverage of SMA criminalisation.

## Plain language summary

Having an abortion at home without a healthcare provider (also known as self-managed abortion or SMA) is common in the United States. Although SMA is legal in nearly every state, the number of people who have been arrested for alleged SMA has continued to grow in recent years. Reproductive justice advocates have identified media coverage as a major influence on how the public views people facing criminal proceedings after SMA; however, researchers have not yet examined this. We analysed 41 news articles on SMA criminalisation published between 2000 and 2023 by the five most popular national newspapers in the United States. Our results show that journalists often (1) portrayed the fetus as a victim who was harmed by the criminalised person, (2) used stories about the criminalised person’s past to portray them as immoral, and (3) repeated police statements that assumed the criminalised person was guilty. These results build on what is known about SMA criminalisation by analysing news media depictions that help to make SMA criminalisation seem normal. Our findings also add further evidence to reproductive justice advocates’ demands for journalists to use better practices when reporting on SMA criminalisation.

## Résumé

L’avortement autogéré est très répandu aux États-Unis d’Amérique et il est devenu de plus en plus fréquent depuis l’arrêt de la Cour suprême de 2022 dans l’affaire Dobbs v. Jackson Women’s Health Organization, qui a permis aux États d’interdire les avortements précoces. Même si l’avortement autogéré est légal dans presque tous les États américains, le nombre de personnes arrêtées pour participation présumée à un avortement autogéré n’a cessé d’augmenter ces dernières années. Les défenseurs de la justice reproductive ont identifié la représentation médiatique de ces cas comme un facteur susceptible de contribuer à des conséquences juridiques défavorables pour les personnes criminalisées; néanmoins, les chercheurs n’ont pas encore étudié cette question. Par conséquent, nous souhaitions évaluer de manière qualitative la manière dont les médias d’information nationaux présentent les cas de criminalisation de l’avortement autogéré. Nous avons identifié 41 articles publiés par les cinq principaux journaux nationaux entre 2000 et 2023 et mené une analyse thématique réflexive. Notre analyse a révélé des tendances préoccupantes dans la couverture médiatique. En effet, les journalistes décrivaient fréquemment le fœtus comme une victime tout en critiquant la personne criminalisée, ternissaient sa réputation à travers des histoires personnelles choisies et encourageaient la présomption de culpabilité en traitant les déclarations de police biaisées comme des comptes-rendus objectifs. Ces résultats enrichissent les publications en décrivant les manières dont l’injustice reproductive est légitimée par les médias d’information et apportent un soutien empirique aux appels des défenseurs de la justice reproductive en faveur d’un journalisme plus responsable dans la couverture médiatique de la criminalisation de l’avortement autogéré.

## Resumen

La autogestión del aborto (AGA) es prevalente en Estados Unidos (EE. UU.) y se ha vuelto cada vez más común desde la decisión de la Corte Suprema en el caso de 2022 de *Dobbs c. Jackson Women’s Health Organization*, que permitió a los estados promulgar la prohibición del aborto temprano. Aunque la AGA es legal en casi todos los estados de Estados Unidos, la cantidad de personas que han sido arrestadas por su supuesta participación en AGA ha continuado aumentando en los últimos años. Promotores de la justicia reproductiva han identificado representaciones de estos casos en los medios de noticias como un probable contribuidor a resultados jurídicos adversos para las personas penalizadas; sin embargo, los investigadores aún no han investigado esto. Por ello, nos propusimos evaluar cualitativamente las maneras en que los medios de noticias nacionales representan los casos de penalización de AGA. Identificamos 41 artículos publicados por los cinco principales periódicos nacionales entre 2000 y 2023 y realizamos un análisis temático reflexivo. Nuestro análisis reveló patrones preocupantes en la cobertura por los medios de noticias, en que los periodistas frecuentemente personificaban al feto como víctima a la vez que vilipendiaban a la persona penalizada, menoscababan el carácter de la persona penalizada por medio de historias personales seleccionadas y promovían la presunción de culpabilidad al tratar declaraciones policiales sesgadas como informes objetivos. Estos hallazgos contribuyen a la literatura al describir las maneras en que los medios legitiman la injusticia reproductiva, y ofrecen apoyo empírico a los promotores de la justicia reproductiva en sus llamados a un periodismo más responsable en la cobertura mediática de la penalización de la AGA.

## Introduction

Unlike clinician-managed abortion care that is provided in a clinical facility or through telehealth services, self-managed abortion (SMA) refers to the process by which a pregnant person ends a pregnancy at home without the direct involvement of a clinician.^1,2^ SMA is common in the United States (US), and research indicates that its use has only grown since the 2022 US Supreme Court ruling in *Dobbs v. Jackson Women’s Health Organization* (*Dobbs*) that eliminated federal protections for abortion and has resulted in more than twenty US states enacting total or early bans on clinician-managed abortion.^3–5^ The cost and overall inaccessibility of clinician-managed care across the US are frequently cited reasons for self-managing an abortion, but many people also choose SMA based on preferences regarding privacy, support, comfort, and de-medicalisation of care.^6–8^ By the letter of the law, SMA is legal in every US state except one – but this has not been the reality for a growing number of people across the country who have faced charges.^2^

SMA has historically been discussed in relation to negative health outcomes due to the prevalence of less safe methods (e.g. use of physical trauma, insertion of sharp objects or caustic substances into the uterus, consumption of toxic substances) prior to the legalisation of abortion in the US, when safer alternatives were less accessible or not yet available.^9^ However, the contemporary use of mifepristone and misoprostol pills or misoprostol pills alone for SMA has all but eliminated the potential for significant health risks.^10^ An extensive body of literature has demonstrated that SMA using medication has the same high rates of safety and effectiveness as clinician-managed medication abortion, which also utilises mifepristone and misoprostol.^11,12^ Rather than being prescribed these medications by a licensed clinician practising within their state, a pregnant person who chooses to self-manage an abortion self-sources them, often through online pharmacies, community networks, or out-of-state telehealth services.^4^ In addition to this rapidly growing use of an exceedingly safe method for SMA, a number of organisations, such as Self-Managed Abortion Safe and Supported (SASS), Plan C, the M + A Hotline, Repro Legal Helpline, Reprocare, and the Repro Legal Defence Fund, also now exist to provide logistical, medical, emotional, and legal support to people who decide that SMA is the best abortion method for their circumstances.^2,10^

Today, it is criminalisation that has emerged as the foremost threat to people who opt for SMA. SMA is currently legal in every US state except one (Nevada, which prohibits SMA after 24 weeks), and only six other states (Arizona, Delaware, Idaho, Oklahoma, New York, and South Carolina) have recently had a SMA ban in place.^13^ Despite this widespread legality, researchers have documented at least 61 instances across 26 states between 2000 and 2020 where an individual was criminally investigated or arrested for allegedly self-managing an abortion or assisting another person in doing so.^13^ Black, Hispanic, and low-income people were disproportionately represented among those criminalised, and 83% of individuals who were ultimately prosecuted were charged not through an SMA ban but through a variety of misapplied charges, such as child abuse, assault, practising medicine without a licence, fetal harm, and homicide. The researchers also acknowledged that the true number of these cases is likely to be much higher, given that their research was limited by the difficulty of identifying cases with misapplied charges and by non-responses to record requests submitted to state administrative offices, courts, and departments of corrections.^13^

Unfortunately, the increasing criminalisation of SMA is also part of a growing trend of criminalising other pregnancy-related health outcomes, such as a pregnant person’s refusal of medical care, substance use during pregnancy, or experiencing a miscarriage or stillbirth.^14–16^ The Reproductive Justice Framework – a paradigm developed by twelve Black women in 1994 to centre the intersectional reproductive needs and experiences of Black women and other marginalised people – emphasises bodily autonomy as an overarching human right, one that encompasses the right to have children, the right to not have children, and the right to live and parent in safe and healthy environments.^17,18^ The criminalisation of SMA and other pregnancy outcomes violates each of these essential rights. Whether enacted through incarceration, deportation, or the family regulation system, SMA criminalisation leverages fear, stigma, surveillance, and punishment to constrain reproductive agency and penalise self-care.^19^ Through this oppressive web of criminalisation, people are separated from their families, deprived of basic needs, and subjected to various other forms of state violence, which simultaneously produces a chilling effect that leads many pregnant people to feel that they have no choice but to continue undesired or mistimed pregnancies.^20–23^ This is particularly true for populations that have historically been targeted by carceral systems in the US, including Black and brown people, immigrants, people with fewer economic resources, disabled people, and transgender and queer people.^19^

As more and more people come to rely on SMA as a means of exercising bodily autonomy in the post-*Dobbs* era, it has only become more urgent that policymakers at all levels take action to decriminalise pregnancy outcomes.^4^ However, policy is not the only way that criminalisation is enacted. Reproductive justice advocates have identified news media portrayals of criminalisation cases as a likely contributor to the broader normalisation of criminalisation and to adverse legal outcomes for criminalised individuals. In particular, the reproductive justice organisations We Testify and the Repro Legal Defense Fund have emphasised the dangers of highly stigmatising articles about individuals who experience SMA criminalisation and their role in entrenching presumptions of guilt.^24–26^ Moreover, decades of research have also shown that framing strategies employed by journalists in pre-trial publicity can prejudice prosecutors, jurors, and judges in ways that lead to more cases brought to trial, more guilty verdicts, and harsher sentences.^27,28^ However, no formal analysis has investigated news media coverage of individuals who have been criminalised in relation to alleged SMA, and little is known about how narratives of state intervention in reproduction are constructed through popular media. Therefore, we aimed to fill these critical gaps by qualitatively assessing the ways in which national news media outlets in the US depict instances of SMA criminalisation.

## Methods

### Data sources

We first identified the top five national newspapers by audience as determined by the Alliance for Audited Media, which found that *The Wall Street Journal*, *The New York Times*, *The Washington Post, USA Today*, and the *Los Angeles Times*, respectively, reached the largest audiences.^29^ We then conducted a search of Pro-Quest databases for articles in these five newspapers that reported on instances of criminalisation related to SMA. We tested several variations of our query to optimise our search terms and obtain the most relevant articles, with final search terms consisting of the following: “self-managed abortion”, “self-induced abortion”, “illegal abortion”, “alleged abortion”, “abortion pill”, mifepristone, misoprostol, arrest, charge, indict, jail, investigate, and guilty. Articles were included if they (1) were published online or in print between January 2000 and December 2023; (2) focused primarily on one or more instances of investigation, arrest, or prosecution associated with allegedly self-managing an abortion or assisting another person in doing so; and (3) were available online for review. Given the variation in laws and legal systems internationally, we excluded any articles that appeared in these US-based outlets but referred to an instance of SMA criminalisation occurring outside the US. Our search yielded a total of 938 results. After removing duplicate articles and systematically screening all articles for relevance, we identified 41 articles that met all inclusion criteria. We downloaded the full text of each article for coding and analysis.

### Coding and analysis

To identify patterns of meaning within this textual data, we conducted reflexive thematic analysis in accordance with Braun and Clarke’s iterative six-phase process and the interpretive paradigm.^30^ Our coding team consisted of two public health students with graduate-level training in qualitative methods and expertise in abortion access and relevant US policies. To familiarise ourselves with the data, each coder independently reviewed and annotated all articles (*N* = 41). We then convened to develop an initial codebook of deductive codes based on the existing literature (e.g. *Police Statement*, *Gestation, Alleged Method*) and inductive codes drawn from initial patterns we identified within our memos (e.g. *Fetal Remains*, *Character Statement, Adversarial Relationship*). After refining the codebook through an intercoder reliability exercise, all data were systematically coded using MAXQDA qualitative software. The analysis team met regularly throughout the process to practice reflexivity, assess code and meaning saturation, and discuss potential themes. We used MAXQDA code reports to collaboratively develop our initial themes through cross-case comparisons and code memos. Finally, we iteratively refined and defined these themes using data displays and theme mapping.^30,31^ Throughout this process, we also validated themes through researcher triangulation by comparing results from multiple analysts and through concept-indicator modelling, a tool borrowed from Grounded Theory that involves making constant comparisons between salient concepts and the raw data to confirm themes are well-grounded.^31,32^

### Ethical considerations

Prior to conducting any research activities, we used the Emory University Institutional Review Board (IRB) Non-Human Subjects Determination tool to ascertain that this study did not involve human subjects; therefore, it was not subject to IRB review or informed consent procedures. Although all data utilised in this research are publicly available, we intentionally omit the names of people who were criminalised to prevent further harm and stigmatisation that could result from their names being publicised again. Instead, we use the state and year of the case to identify individual instances of criminalisation.

### Positionality

While we have sought to practice reflexively and bracket our assumptions throughout this analysis, the interpretive paradigm emphasises that all research is inherently shaped by the intersecting identities and lived experiences of the researchers.^31^ Therefore, we wish to offer transparency in the positionalities of our authorship team. Five authors identify as cisgender women, and two authors identify as non-binary women. Regarding race-ethnicity, three authors identify as Black, one author identifies as Black and South Asian, and three authors identify as white. At the time of analysis and drafting of the manuscript, two authors were master’s students, two authors were doctoral students, and three authors were university faculty holding the rank of assistant professor. Six authors resided in an abortion-restrictive state in the US Southeast, and one author resided in an abortion-supportive state on the US West Coast. Relevant areas of expertise among members of the authorship team include sexual and reproductive health, communications, structural racism, policing and carceral systems, bioethics, feminist theory, health equity, law and policy, and qualitative methodology.

## Results

The characteristics of the included articles (*N* = 41) are summarised in Table 1, and detailed information for all included articles and identifier codes are available in Appendix A. The greatest proportion of articles was published in *The Washington Post* (36.6%, *n* = 15), followed by *The New York Times* (26.8%, *n* = 11). More than half of the articles were published between 2016 and 2022 (56.1%, *n* = 23). Our sample included news coverage of 11 different instances of SMA criminalisation. One instance of SMA criminalisation in Texas in 2022 and one in Tennessee in 2015 were the most widely covered, with seven articles (17.1%) published per case. We organised our findings into three overarching themes: *Creating a Victim and a Villain by Personifying the Fetus*, *Undermining Character by Exploiting Personal Histories,* and *Presuming Guilt by Treating Police Statements as Objective Accounts*.

**Table 1.** Summary of sample characteristics (*N* = 41)

Characteristic	Proportion (*n*)
**News Outlet**	
*The Washington Post*	36.6% (15)
*The New York Times*	26.8% (11)
*USA Today*	17.1% (7)
*The Wall Street Journal*	9.8% (4)
*Los Angeles Times*	9.8% (4)
**Year of Publication**	
2000–2007	7.3% (3)
2008–2015	36.6% (15)
2016–2023	56.1% (23)
**Case Referenced in Article**	
Maryland 2007	7.3% (3)
New York 2011	7.3% (3)
Idaho 2012	2.4% (1)
Pennsylvania 2014	4.9% (2)
Indiana 2015	12.2% (5)
Georgia 2015	12.2% (5)
Tennessee 2015	17.1% (7)
Virginia 2015	4.9% (2)
Texas 2022	17.1% (7)
Nebraska 2022	12.2% (5)
South Carolina 2023	2.4% (1)

### Creating a victim and a villain by personifying the fetus

News articles frequently contained vivid depictions of fetal remains, which are often used as evidence in cases against people who experience SMA criminalisation.^13^ The personifying detail in descriptions of these fetal remains further affirmed characterisations of the fetal remains as a “baby,” “infant,” or “child” – terms that imply legal personhood. In emphasising this, news articles often included the approximate gestation of the pregnancy, as well as sometimes specifying weight, sex, and other anatomical markers of development. For example, in coverage of a case from Maryland in 2007, *The Washington Post* reported the following:

*Increasingly gruesome details of the case emerged throughout the day … In a vanity under the bathroom sink, [police] discovered a 26-week-old, 2 ½-pound stillborn child wrapped in a blood-smeared towel … authorities said the stillborn child … had the ‘basic structures of a human’ – eyes, nose, mouth, torso, arms, hands and fingers, legs, feet and toes. The child appeared to be a boy*. (Article MD07WPO1)

Similarly, sensationalised narratives depicting the discovery of fetal remains and the methods of disposal underscored the alleged carelessness of the person who was criminalised, as seen in a news article from *The New York Times* about a 2011 case from New York:

*[The criminalised person] was charged with self-abortion in the first degree on Wednesday, two days after the lifeless male fetus was found in a trash can at her [neighborhood] building. The body had been placed in a bucket and wrapped in black plastic bags … The case began Monday evening after [the building superintendent and his son] made the grim discovery while sorting the trash*. (Article NY11NYT1)

In contrast to the extensive, personifying detail that was provided to describe fetal remains, the criminalised person was frequently characterised solely by their alleged illegal behaviour; however, some news articles went further in actively dehumanising them. By presenting the fetus as a vulnerable “baby” and then juxtaposing the disposal of fetal remains with discarded trash, many news articles portrayed the criminalised person as callous and malicious, as exemplified in the following article from *The Washington Post* on a 2015 case from Indiana:

*–[The judge] said that [the criminalised person] was in a position to legally end her pregnancy, but opted for an illegal method, and later “ensured that baby’s death by placing him in the trash can with the other bathroom trash*”. (Article IN15WPO1)

This persistent narrative of an adversarial relationship between the criminalised person and their pregnancy depicted the criminalised person as abnormally detached and inhumane, seemingly lacking the implied natural inclination of women towards motherhood. In a *New York Times* article about the 2015 case from Tennessee, one journalist published a particularly disparaging quote from a police spokesperson:

*“She wanted to kill the child,” said [the police spokesman]. In the hospital, he said, [the criminalised person] “made very incriminating statements, and very concerning statements … The whole time she was concerned for her health, her safety and never gave any attention to the safety of the unborn child”.* (TN15NYT1)

Throughout the news articles, the systematic dehumanisation of the criminalised person as a heartless villain and the personification of the fetus as a vulnerable “child” created a pervasive narrative of the existence of two distinct individuals involved in SMA, with the fetus repeatedly cast as the victim of an alleged crime.

### Undermining character by exploiting personal histories

As evidenced in the above quotation, the character of the criminalised person was also systematically undermined through the insertion of purported details from their past that portrayed them as immoral and irresponsible. Selected personal histories associated with other highly stigmatised experiences, such as substance use, miscarriage, incarceration, and mental illness, were frequently highlighted, even when these details were irrelevant to the case. For instance, in covering a 2015 case from Virginia, a journalist at *The Washington Post* included the criminalised person’s alleged history of substance misuse with no apparent connection to the case:

*According to the police, [the criminalised person] told investigators she gave birth to a baby boy in the house, but he died afterward, and she buried him in the back yard. Police and prosecutors have revealed little about the evidence that led to the unusual charge against [the criminalised person], who court records show has a history of drug use. (VA17WPO1)*

Another article from the *Los Angeles Times* highlighted a quote from the prosecutor in a 2012 case in Idaho, who utilised the criminalised person’s prior pregnancies that ended in miscarriage or clinician-managed abortion as evidence of her alleged carelessness:

*This wasn't the first time this has happened. She's had abortions before, and miscarriages. I mean, she was obviously getting pregnant time and time again and not protecting the unborn fetus*. (ID12LAT1)

Likewise, condemning quotes from family and neighbours of the criminalised person also often highlighted prior interactions with the carceral system or potential indicators of mental illness, thereby suggesting a pattern of indecent or erratic behaviour. Most claimed a personal relationship with the criminalised person, but then quickly distanced themselves, emphasising the unacceptability of the criminalised person’s alleged actions. In regard to a 2015 case in Georgia, *The Washington Post* reported the following:

*[The criminalised person] has always been troubled, [the brother of the criminalised person] said. Her life was riddled with run-ins with the law, he said, and psychological strain that was never diagnosed * … * “At the end of the day, she is mentally unstable. It doesn't make it right to take a life too, but still.”* (GA15WPO1)

These journalistic choices consistently depicted the criminalised person as someone with a history of moral shortcomings and exploited the stigma associated with substance use, miscarriage, incarceration, and mental illness to reinforce social biases. At the same time, these narratives also contributed to the broader stigmatisation of people who self-manage an abortion by portraying SMA as nefarious and presenting criminalisation as a reasonable consequence.

### Presuming guilt by treating police statements as objective accounts

Articles generally neglected the perspective of the person experiencing criminalisation, with most articles lacking any statement from criminalised individuals or their legal representatives. Instead, journalists predominantly relied on statements from police and prosecutors, which were frequently presented as objective truths, despite their inherent bias. In contrast to a constitutionally guaranteed presumption of innocence, police consistently provided public statements asserting the criminalised person’s guilt as a matter of fact, and these presumptions were indiscriminately reproduced in many news articles. Writing about a Tennessee case that began in 2015, for instance, a journalist at *The Washington Post* uncritically reported this allegation from police, in which they described a dramatised narrative of a highly specific, step-by-step process that they claimed was used by the criminalised person to end their pregnancy:

*[The criminalised person] was 24-weeks pregnant when police say she filled a bathtub with water, untwisted the wire of a coat hanger and plunged it into her womb in an attempt at “self abortion”.* (TN15WPO1)

Similarly, a *Los Angeles Times* journalist failed to interrogate police’s outright rejection of the statement made by a person who was criminalised in Idaho in 2012, where police dismissed her words as “obviously” incorrect, an assertion evidenced only by a non-expert opinion on fetal development:

*[The criminalized person] believed she was only about 14 weeks pregnant … Police say the fetus was obviously much older than 14 weeks. It had fully formed facial features, tiny fingernails, hair*. (ID12LAT1)

This was also observed in reporting of police statements about the severity of the alleged crime, as seen in a *New York Times* article about a 2022 case from Nebraska, in which the journalist included asides from police that appeared to cast unnecessary doubt on the official findings of a medical examiner:

*The remains were exhumed and showed signs of ‘thermal injuries,’ the detective wrote … often the biggest question is whether the baby was born alive. If so, the woman involved can face more serious charges. In the [Nebraska 2022] case, the final autopsy listed the cause of death as undetermined. “The findings were consistent with the fetus being stillborn”, but the fetus had been put in a plastic bag, raising the possibility of suffocation, the detective wrote*. (NE22NYT1)

Furthermore, journalists frequently noted that police statements were based on information provided to them by healthcare workers who had initially reported the individual facing criminalisation: *“[The criminalised person] was arrested Saturday after a [hospital] social worker told the police that she had taken four [misoprostol] pills she ordered online”* (GA15WPO). By highlighting healthcare workers’ active participation in surveillance and policing, additional credibility was lent to biased police narratives: “*Police said doctors and nurses who treated [the criminalised person] told them that she had wanted to terminate the pregnancy”*. (TN15WPO)

In emphasising these instances of healthcare workers acting as police proxies, journalists also provided misleading or false information about healthcare workers’ purported obligation to report SMA to the police. Notably, we identified only one article that accurately stated that healthcare workers are not legally required to report SMA in any state, while several others erroneously suggested or even explicitly stated otherwise.^33,34^ For example, *The Washington Post* reported the following regarding a 2015 case from Indiana:

*[A hospital physician], who is obligated to report cases of suspected child abuse, called the police, he told [a local news outlet]. Informed that officials were heading to her home, [the criminalized person] told her doctors that she’d had a miscarriage and had left her stillborn fetus in a dumpster behind a shopping center. Still in his hospital scrubs, [the physician] followed police cars to the scene and examined the fetus, which he pronounced dead on arrival*. (IN15WPO1)

This article uncritically validated the false notion that alleged SMA constitutes the crime of “child abuse,” thereby endorsing the physician’s inaccurate claim that they are required by law to report alleged SMA. Across articles, statements from police and prosecutors – often corroborated by healthcare workers acting as police proxies – were the primary source of information used in reporting on instances of SMA criminalisation. An obvious bias exists in these narratives, given that police and prosecutors are the very parties pursuing charges against an individual facing criminalisation, yet most articles failed to question the presumptions of guilt or justifications of the charges.

## Discussion

The realisation of Reproductive Justice requires that we analyse systems of power and the intersecting ways in which they shape reproductive politics and health.^35^ While the growing criminalisation of SMA and other pregnancy outcomes in the US is primarily driven by carceral policymaking, it is clear that policing and prosecution are not the only ways that this criminalisation is enacted.^19^ By qualitatively exploring depictions of SMA criminalisation in news media, this study offers an empirical analysis of a largely unexplored mechanism through which reproductive autonomy is constrained. Journalists, as actors within a major sociopolitical institution, have immense power in shaping societal narratives about SMA and pregnancy more broadly, but it is a power that must be wielded thoughtfully to avoid normalising criminalisation and legitimising stratified reproduction.

At the individual level, it has been well established that media representations are influential in criminal investigations and prosecutions. Prior research has demonstrated that negative media portrayals are associated with prosecutors pursuing more severe charges, jurors reaching more guilty verdicts, and judges imposing harsher sentences.^27,28^ Undoubtedly, the manner in which national news articles have negatively portrayed people who are criminalised for alleged SMA can have extensive implications for these individuals’ lives and wellbeing. By personifying fetal remains with extensive detail and framing the fetus as a victim and SMA as a crime cruelly perpetrated by a person who was pregnant, news media articles elevated the legal status of the fetus and vilified individuals experiencing criminalisation.^14^ Their inclusion of selected information about the criminalised individual’s personal history further exploited the harmful stigma that is tied to substance use, miscarriage, incarceration, and mental illness to reinforce social biases and characterise these individuals as fundamentally irresponsible and unfit for pregnancy or parenting.^14,15,36–39^ Moreover, despite the extensive evidence demonstrating the high prevalence of falsified information in arrest reports, warrants, and courtroom testimony, police narratives that sought to justify a carceral response to pregnancy outcomes were overwhelmingly regarded as objective accounts throughout news media coverage of SMA criminalisation.^40,41^ Journalists’ ubiquitous use of these statements made by police and self-deputised healthcare workers served to further normalise criminalisation, reinforce inaccurate beliefs about mandatory reporting practices, and exacerbate the distrust that deters others from seeking care for SMA complications that are rare but not impossible.^11^ Irrespective of whether these stigmatising portrayals of SMA in news media are intentional, they play a significant role in shaping public perceptions and have the power to profoundly harm people who experience criminalisation. Even in instances when a criminalised person’s charges are ultimately dropped, accusations that are often uncritically publicised by journalists mark people with a stigma that has reverberating effects.^13^ In articles that covered cases where charges were dropped or appeals were won, we noted that several people who experienced criminalisation explicitly stated that they had still lost their jobs or that they and their families were still isolated from their community because of the allegations. It is imperative that journalists heed the warnings of reproductive justice advocates to think critically about the language used to portray people facing SMA criminalisation and prioritise ethical reporting practices over stigma and sensationalism.^24–26^

Beyond these harmful implications for individuals targeted by SMA criminalisation, the narratives we examined are also indicative of concerning trends in societal conceptualisations of pregnancy and state involvement in regulating reproduction. Journalists frequently depicted undesired or mistimed pregnancies as adversarial relationships between two distinct individuals, thereby normalising the extremist concept of “fetal personhood,” an extreme dangerous, far-right legal theory asserting that a zygote is a distinct person entitled to the same legal rights as the pregnant person, and that abortion should be criminalised in all instances.^42–44^ The frequent portrayal of the embryo or fetus as a victim sharply contrasts with the depiction of the criminalised person as callous for allegedly ending their pregnancy, speaking to the ways that abortion through any means is often cast as a deviation from prevailing social norms that presume the innate response of all pregnant people is instinctive protectiveness towards the fetus.^45^ As legal scholar Dorothy Roberts has argued, pregnant people who deviate from this “natural” standard are perceived as a threat to the fetus, thereby fuelling a carceral logic that begets pregnancy criminalisation. This notion of an alleged maternal-fetal conflict experienced among those deemed “unfit” is employed to justify the paternalistic surveillance and policing of pregnancy, wherein the state intervenes under the guise of protecting the personified fetus to exert control over the pregnant person and impose punitive measures for alleged harms.^23,38^ By not only reflecting but also reinforcing this paradigm, news media representations normalise state interference in bodily autonomy during pregnancy.

Importantly, this designation of who is “unfit” to reproduce is not uniformly applied across society but is instead asymmetrically directed at the most marginalised. This analysis offers a snapshot of SMA criminalisation as a continuity in the ongoing history of stratified reproduction in the US, one in which public policy and media narratives converge to reinforce hegemonic ideals of who should and should not reproduce.^37,39^ The US, driven by white supremacist ideologies, has historically leveraged law and social norms to discourage reproduction among people of colour and low-income people, while simultaneously promoting reproduction among white, able-bodied people with financial resources.^39,46^ Our findings, in alignment with Maura Kelly’s previous analysis of the “controlling image” of Black and low-income “welfare mothers” on televised news during US welfare reform, demonstrate how policies that allow state intervention in reproduction among the “unfit” are popularised through news media that characterise people who are marginalised by structural racism, systemic poverty, and other systems of oppression as a danger to their own progeny.^47^ We observed that people criminalised for alleged SMA – people who are predominantly Black, brown, and low-income – were consistently depicted in news media as a threat to the personified fetus, thereby positioning the state as a benevolent protector rather than an intrusive force in individuals’ bodily autonomy. As with anti-abortion policies more generally, considering the realities of criminalisation reveals how the denial of abortion to populations whose reproduction has historically been deemed undesirable serves the purposes of stratified reproduction.^19,46^ Through criminalisation and incarceration, the state can fully dominate individuals’ reproductive capacities in ways that have been largely accepted by society.^37^ The carceral system explicitly denies the fundamental human rights articulated by the Reproductive Justice Framework. Incarcerated individuals are systematically deprived of bodily autonomy, the ability to plan desired pregnancies, the means to prevent or end undesired pregnancies, the opportunity to parent their children, and the capability to live in a safe and healthy environment.^37,48^ Criminalisation in all its forms, though particularly in the criminalisation of pregnancy outcomes, perpetuates a cycle of marginalisation and reproductive injustice, further entrenching stratified reproduction.

### Limitations and future directions

This research should be interpreted within the context of several limitations. First, articles from local or regional news outlets were not included. We identified many examples of local and regional news reporting on SMA criminalisation during our search; however, these articles were outside the scope of this study. It is possible that cases of SMA criminalisation are portrayed differently in these localised outlets. The next step of this research will be to identify a sample of regional and local news articles to further examine localised news reporting on SMA criminalisation. Second, this study does not encompass reporting on all known instances of SMA criminalisation that occurred between 2000 and 2023. Although we conducted an extensive search and reviewed more than 900 potentially relevant articles, the 41 articles that met our inclusion criteria covered only 11 unique instances of SMA criminalisation. Prior research has identified at least 61 unique cases of SMA criminalisation between 2000 and 2020.^13^ These other cases may be missing from our sample because they were not reported in news media, were only reported by local and regional media, or were simply not captured by our search terms. In addition, the relatively small number of unique cases, along with the limited availability of self-reported demographic information, prevented us from being able to meaningfully compare reporting across the racial-ethnic identities of criminalised individuals. Existing research on news media coverage of other alleged crimes has extensively documented systemic racial bias, and there is no reason to believe that reporting on SMA criminalisation would be an exception.^49,50^ Future research should examine whether the language and journalistic strategies in news articles differ across the racial-ethnic identity of the criminalised person. Likewise, this sample included only a single instance of criminalisation that occurred after *Dobbs* (some articles that were published in 2022 and 2023 refer to cases that originated prior to *Dobbs*); therefore, we cannot draw conclusions regarding possible shifts in news media representations of SMA following the ruling and the associated coverage of SMA as an increasingly common practice. Sadly, multiple well-publicised instances of SMA criminalisation have already occurred in the first half of 2025, so it is evident that additional research will be needed to investigate potential changes in media narratives over time.^51,52^

## Conclusion

The fact that only one US state prohibits self-managed abortion has not prevented people in 26 states across the country from facing criminalisation and incarceration for allegedly ending their own pregnancies or helping another person do so. Until the explicit decriminalisation of all pregnancy-related health outcomes is realised, there will continue to be a need to mitigate contributing factors. Particularly in the post-*Dobbs* era, as individuals’ ability to exercise bodily autonomy has only been further constrained by the growing criminalisation of abortion, it is increasingly necessary that we critically examine the various systems of power that shape reproductive politics. This study examined representations of self-managed abortion criminalisation in news media, a prominent social institution that plays a pivotal role in shaping public opinion and likely exerts considerable influence on the outcomes of individual cases of criminalisation and on broader societal perceptions of pregnancy policing. Throughout this analysis, we identified concerning patterns in news media coverage of self-managed abortion criminalisation, wherein journalists frequently personified the fetus as a victim of the criminalised person’s alleged actions, undermined the character of the criminalised person by exploiting personal histories, and promoted the presumption of guilt by treating biased police narratives as objective facts. These findings provide further empirical support for reproductive justice advocates’ calls for responsible journalism that centres the harm to the individual experiencing criminalisation, rejects publication of the criminalised person’s name or photograph whenever possible, exercises caution when reporting personal histories, questions the validity of the charges, and provides clear and accurate information about the legality of SMA.^24–26^ It is imperative that journalists wield the power of the media thoughtfully to avoid legitimising the carceral logic that underpins pregnancy criminalisation and its manifestations through both courts of law and the court of public opinion.

## Author contributions

*Conceptualisation: HVM, WSR. Data curation: HVM, NP. Formal analysis: HVM, NP. Supervision: SKR, WSR. Funding acquisition: WSR. Writing - original draft: HVM, NP. Writing - review & editing: HVM, AJS, ICS, GEH, SKR, WSR*.

## Data Availability

All data utilised in this study are publicly available.

## Provenance statement

This article was not commissioned and went through external peer review.

## Appendix A. Included articles and corresponding identifiers (*N* = 41)

**Table:**

Identifier	Case	Article information
MD07NYT1	Maryland 2007	Associated Press. Bodies of 4 infants found at Maryland home. *The New York Times*. July 31, 2007.
MD07WPO1	Maryland 2007	Morse D, Wan W. Mother charged in stillborn death. *The Washington Post.* July 31, 2007.
MD07WPO2	Maryland 2007	Wan W. New murder charge replaces original in case against mother. *The Washington Post.* August 3, 2007.
NY11NYT1	New York 2011	Hartocollis A. After fetus is found in trash, a rare charge of self-abortion. *The New York Times.* December 2, 2011.
NY11WSJ1	New York 2011	Gardiner S. Rare charge: Self-abortion. *The Wall Street Journal.* December 2, 2011.
NY11WSJ2	New York 2011	Shallwani *P*. Abortion charge dismissed. *The Wall Street Journal.* January 4, 2012.
ID12LAT1	Idaho 2012	Murphy K. Idaho woman’s case marks a key abortion challenge. *Los Angeles Times.* June 16, 2012.
PA14WPO1	Pennsylvania 2014	Reuters. Mom imprisoned for buying teen daughter abortion pills online. *The Washington Post.* September 7, 2014.
PA14NYT1	Pennsylvania 2014	Bazelon E. A mother in jail for helping her daughter have an abortion. *The New York Times.* September 22, 2014.
IN15WPO1	Indiana 2015	Kaplan S. Indiana woman jailed for “feticide.” It’s never happened before. *The Washington Post.* April 1, 2015.
IN15USA1	Indiana 2015	Disis J. Ind. woman’s sentence for self-abortion draws scrutiny. *USA TODAY.* May 3, 2015.
IN15WPO2	Indiana 2015	Callahan R. Appeal likely in feticide conviction. *The Washington Post.* May 22, 2016.
IN15LAT1	Indiana 2015	Callahan R. Abortion appeal case is heard. *Los Angeles Times.* May 24, 2016.
IN15WPO3	Indiana 2015	Wootson Jr CR. Court overturns feticide conviction of Indiana woman who had self-induced abortion. *The Washington Post*. July 22, 2016.
GA15WSJ1	Georgia 2015	Associated Press. Murder charge after abortion pill used. *The Wall Street Journal.* June 11, 2015.
GA15WSJ2	Georgia 2015	Associated Press. Murder charge dropped in abortion pill case. *The Wall Street Journal.* June 11, 2015.
GA15LAT1	Georgia 2015	Jarvie, J. Murder charge dropped against Georgia woman who took pills for abortion. *Los Angeles Times.* June 10, 2015.
GA15NYT1	Georgia 2015	Associated Press. Georgia: No murder charge for woman who bought abortion pills online. *The New York Times.* June 10, 2015.
GA15WPO1	Georgia 2015	Phillip, A. Murder charges dropped against Georgia woman jailed for taking abortion pills. *The Washington Post*. June 10, 2015.
TN15NYT1	Tennessee 2015	Eckholm E. Tennessee woman tried coat-hanger abortion, police say. *The New York Times*. December 16, 2015.
TN15WPO1	Tennessee 2015	Kaplan S. Tenn. woman charged with attempted murder for failed coat hanger abortion. *The Washington Post*. December 16, 2015.
TN15USA1	Tennessee 2015	Willard M. New charges filed in Tenn. coat-hanger abortion case. *USA TODAY.* Published November 16, 2016.
TN15WPO2	Tennessee 2015	Mettler K. Tennessee woman who attempted coat hanger abortion faces three new felony charges. *The Washington Post.* November 18, 2016.
TN15NYT2	Tennessee 2015	Hauser C. Tennessee woman accused of coat-hanger abortion attempt faces new charges. *The New York Times*. November 29, 2016.
TN15NYT3	Tennessee 2015	Stack L. Woman accused of coat-hanger abortion pleads guilty to felony. *The New York Times.* January 11, 2017.
TN15WPO3	Tennessee 2015	Hawkins D. Tenn. woman pleads guilty to felony in attempted coat hanger abortion. *The Washington Post*. January 12, 2017.
VA17WPO1	Virginia 2017	Brown DL. “I know what’s buried in the back yard”: A woman faces a rare charge of self-induced abortion. *The Washington Post.* April 20, 2017.
VA17WPO2	Virginia 2017	Brown DL. Va. prosecutors dismiss case against woman accused of “self-aborting.” *The Washington Post*. October 5, 2018.
TX22WPO1	Texas 2022	Torbati Y, Kitchener C. Texas woman charged with murder after abortion. *The Washington Post.* April 9, 2022.
TX22USA1	Texas 2022	Fernando C. Woman faces murder charge in Texas after “self-induced abortion,” prompting outrage from reproductive rights advocates. *USA TODAY.* April 10, 2022.
TX22NYT1	Texas 2022	Heyward G, Kasakove S. Texas will dismiss murder charge against woman connected to “self-induced abortion.” *The New York Times.* April 10, 2022.
TX22WPO2	Texas 2022	Kitchener C. Murder charges to be dropped for Texas woman arrested over abortion. *The Washington Post.* April 10, 2022.
TX22USA2	Texas 2022	Stanton C. Texas judge dismisses murder charge in “self-induced” abortion case due to insufficient evidence. *USA TODAY*. April 10, 2022.
TX22WPO3	Texas 2022	Kitchener C, Reinhard B, Crites A. Woman’s arrest stirs ire on both sides of abortion debate. *The Washington Post.* April 14, 2022.
TX22USA3	Texas 2022	Kenning C. A Texas woman’s murder charge for a “self-induced abortion” was dropped. Why the fallout may linger. *USA TODAY.* April 15, 2022.
NE22NYT1	Nebraska 2022	Dewan S, Frenkel S. A mother, a daughter and an unusual abortion prosecution in Nebraska. *The New York Times.* August 18, 2022.
NE22NYT2	Nebraska 2022	Levenson M. Nebraska teen who used pills to end pregnancy gets 90 days in jail. *The New York Times.* July 20, 2023.
NE22USA1	Nebraska 2022	Perez K. 18-year-old Nebraska woman sentenced to 90 days in jail for burning, burying aborted fetus. *USA TODAY.* July 21, 2023.
NE22LAT1	Nebraska 2022	Beck MA. Nebraska mother sentenced in abortion case. *Los Angeles Times.* September 25, 2023.
NE22NYT3	Nebraska 2022	Jiménez J. Mother who gave abortion pills to teen daughter gets 2 years in prison. *The New York Times.* September 22, 2023.
SC23USA1	South Carolina 2023	Fernando C. South Carolina woman’s arrest draws attention to criminalization of self-managed abortions. *USA TODAY*. March 3, 2023.
